# Increased Surgical Delays Seen During the COVID-19 Pandemic in a Regional Referral Hospital in Soroti, Uganda: Perspective from a Low-Resource Setting

**DOI:** 10.1007/s00268-023-06965-y

**Published:** 2023-03-12

**Authors:** Savannah Starr, Rasheedat Oke, Silas Okullu, Mary Goretty Ariokot, Andrew Hyginus Wange, Esther Agwang, Peter Ekuchu, Catherine Juillard, Mary Margaret Ajiko, Rochelle A. Dicker

**Affiliations:** 1 David Geffen School Geffen School of Medicine, University of California at Los Angeles, Los Angeles, CA USA; 2 Program for the Advancement of Surgical Equity, Department of Surgery, University of California, 10833 Le Conte Avenue, 72160 CHS, Los Angeles, CA 90095 USA; 3 Department of Surgery, Soroti Regional Referral Hospital, Soroti, Uganda

## Abstract

**Introduction:**

The impact of COVID-19 on low-resource surgical systems is concerning but there are limited studies examining the effect in low- and middle-income countries. This study assesses changes in surgical capacity during the COVID-19 pandemic at Soroti Regional Referral Hospital, a tertiary healthcare facility in Soroti, Uganda.

**Methods:**

Patients from a prospective general surgery registry at SRRH were divided into cohorts admitted prior to the pandemic (January 2017 to February 2020) and during the pandemic (March 2020 to May 2021). Demographics, pre-hospital characteristics, in-hospital characteristics, provider-reported delays in care, and adverse events were compared between cohorts.

**Results:**

Of the 1547 general surgery patients, 1159 were admitted prior to the pandemic and 388 were admitted during the pandemic. There was no difference in the median number of elective (24.5 vs. 20.0, *p *value = 0.16) or emergent (6.0 vs. 6.0, *p* value = 0.36) surgeries per month. Patients were more likely to have a delay in surgical care during the pandemic (22.6% vs. 46.6%, *p* < 0.01), particularly from lack of operating space (16.9% vs. 46.3%, *p* < 0.01) and lack of a surgeon (1.6% vs. 4.4%, *p* < 0.01). Increased proportion of delays in care appear correlated with waves of COVID-19 cases at SRRH. There were no changes in rates of adverse events (5.7% vs. 7.7%, *p* = 0.18).

**Discussion:**

The COVID-19 pandemic caused significant increases in surgical care delays and emergency surgery at SRRH. Strengthening surgical systems when not in crisis and including provisions for safe, timely surgical delivery during epidemic resource allocation is needed to strengthen the overall healthcare system.

## Introduction

Since emerging in December 2019 [1], SARS-Co-V-2 (COVID-19) has had widespread impact on healthcare systems globally. In addition to the disease burden from COVID-19, the pandemic has caused many direct and indirect effects on healthcare delivery, including some yet to unfold. For surgical systems, hospitals have recommended prioritization schemes and reductions in elective surgeries to conserve anesthesiology staff, equipment, and ICU beds to manage COVID-19 burden [2]. Though many hospitals saw reductions in emergency surgery services [3–5], emergent surgical conditions persisted, overstretching intensive care resources needed for COVID-19 patients [2].

Given the impact of COVID-19 on robust healthcare systems in high-income countries (HICs), the impact on low-and-middle income countries (LMICs) is a substantial concern but largely unknown. Even prior to the pandemic, LMICs faced an urgent need to build capacity for safe and accessible surgical care. Addressing the unmet need was estimated to require 2.2 million additional surgeons, obstetricians, and anesthesiologists as well as significant investments in surgical equipment and infrastructure [6]. With LMIC surgical systems already under-resourced, strain from COVID-19 may be substantial and hinder progress that has already occurred.

Uganda is a country in sub-Saharan Africa with an estimated 3.6 million people with unmet surgical needs prior to the pandemic [7]. Ugandans requiring surgery face significantly limited operating space [8] and understaffed workforce [9, 10]. The aim of our study is to understand the impact of the COVID-19 pandemic on the surgical landscape at a regional Ugandan hospital.

## Materials and methods

### Study setting

This study was conducted at Soroti Regional Referral Hospital (SRRH), a government-run 250-bed hospital with a catchment area of approximately two million people. It is one of thirteen regional hospitals in Uganda. SRRH has one operating theater with two tables divided by partitions with a staff of two attending general surgeons, two attending gynecological surgeons, and three nurse anesthetists [11]. In addition to being a referral hospital for specialized surgical care, SRRH became the only center in Teso sub-region that managed COVID-19-infected patients.

### Data collection

An ongoing general surgical registry was established at SRRH in 2017. A trained registered nurse served as the registrar who prospectively obtained data on paper forms from patients, providers, and medical records. Then, it was entered into REDCap [12], a secure electronic database hosted at University of California San Francisco (UCSF) and then University of California Los Angeles (UCLA), collaborating institutions with SRRH during the study. Data verification for completeness and accuracy was conducted before and after entry into REDCap. Obstetric and trauma patients were excluded. Collected information included demographics, travel times and methods, length of symptoms, preoperative and postoperative diagnoses, diagnostic laboratory and radiologic studies, operation type, postoperative disposition, reported cause of delay in surgical care, and types of post-surgical complications.

### Analysis

In this prospective study, patients were divided into two study cohorts: those admitted prior to the pandemic and those admitted during the pandemic. Pre-pandemic patients were defined as those admitted January 2017 to February 2020. Pandemic patients were those admitted March 2020 to May 2021.

Demographics and in-hospital characteristics were compared, including total patients per month (excluding May 2021 due to incomplete data), types of operations, and provider-reported delays. Adverse events, a composite variable defined by patients incurring a complication or death, were also compared. Prevalence of delays were observed by month and correlated with internal SRRH COVID-19 data.

Delays were defined by providers caring for each patient, who evaluated times of arrival, decision to operate, and arrival to operating room to determine if patients experienced in-hospital surgical delay, accounting for the procedure and clinical condition of the patient. Providers then reported which factors caused the delay, which was recorded in our registry. Causes of delays were organized into broad categories to represent deficits in personnel, equipment, and infrastructure [13].

Descriptive analyses were presented as medians and interquartile range (IQR) for continuous variables. Categorical variables were presented as frequencies and proportions. Univariate analysis utilized chi-square or Fisher’s exact tests for categorical variables. Continuous variables were analyzed using t test and Mann–Whitney U test for parametric and nonparametric variables, respectively. Data were analyzed using SPSS Version 27 software [14].

### Ethics

Study protocol was approved by SRRH hospital administration and Institutional Review Boards of UCSF and UCLA. Oral informed consent was obtained from patients.

## Results

### Patient characteristics

Of 1547 general surgery patients reported from January 2017 to May 2021, 1159 patients were admitted prior to and 388 were admitted during the pandemic. There was no difference in median age between cohorts (28.5 vs. 24.0, *p* value = 0.09). Pre-pandemic, 56.3% were male while 74.5% were male during the pandemic (*p *value < 0.01) (Table 1). There was no difference in proportion of patients referred from an outside hospital (8.7% vs. 6.2%, *p* value = 0.19). Geographical distribution of patients arriving at SRRH was altered after the onset of the pandemic with a smaller percentage of patients from Amuria (14.6% vs. 7.7%, *p *value < 0.01) and larger percentage from Soroti (51.3% vs. 58.5%, *p* value = 0.03). There was no significant difference in patients from Bukedea, Katakwi, Kumi, Kaberamaido, Serere, or Ngora while other districts not measured by the database had a higher percentage of patients.

**Table 1:** Patient characteristics of general surgery patients at SRRH prior to and during the COVID-19 pandemic (*n* = 1547^a^)

Patient characteristics	Pre-pandemic (*n* = 1159)	Pandemic (*n* = 388)	*p *value
Age (median [IQR]) (*n* = 1543)	28.5 [8.3–49.0]	24.0 [4.0–49.0]	0.09
Male sex (%) (*n* = 1547)	652 (56.3%)	298 (74.5%)	< 0.01*
Referred from outside hospital (%) (*n* = 1524)	101 (8.8%)	24 (6.5%)	0.19
Patient district of residence (travel distance from Soroti) (*n* = 1526)			
Amuria (36 km)	169 (14.8%)	30 (7.7%)	< 0.01*
Bukedea (69 km)	10 (0.9%)	3 (0.8%)	1.00
Katakwi (52 km)	41 (3.6%)	14 (3.6%)	1.00
Kumi (50 km)	42 (3.7%)	14 (3.6%)	1.00
Kaberamaido (73 km)	60 (5.3%)	14 (3.6%)	0.39
Serere (148 km)	114 (10.0%)	41 (10.6%)	0.81
Soroti (0 km)	595 (52.3%)	227 (58.5%)	0.03*
Ngora (47 km)	69 (6.1%)	20 (5.2%)	0.62
Other	38 (3.3%)	25 (6.4%)	0.01*

*p-value < 0.05

^a^Variables had missing data; hence, the total n differs for each variable. Missing data were excluded from the analysis

### Pre-hospital characteristics

**Fig. 1:**
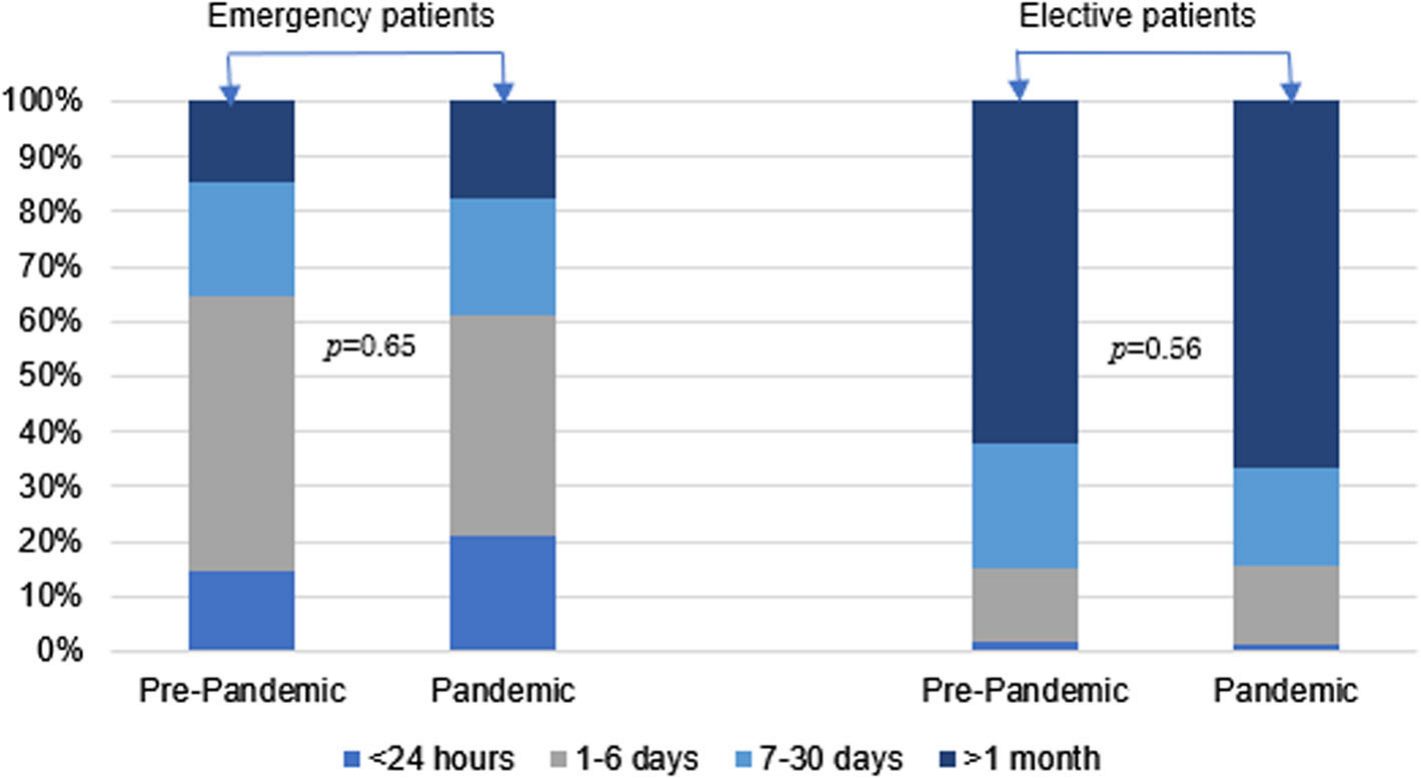
Length of symptoms of emergency and elective general surgery patients arriving to SRRH (*n* = 1527^a^). ^a^Length of symptoms with 4 missing entries for emergent cases and 13 missing entries for elective cases

For both emergent and elective surgical conditions seen at SRRH, there was no difference in length of symptoms prior to arrival to the hospital (Fig. 1). Differences in travel method was seen between cohorts (Table 2). Boda was the most popular transport method between both cohorts and its use increased during the pandemic (47.6% vs. 57.2%, *p *value < 0.01). There was no difference in patients using a bus (0.1% vs. 0.0%, *p* value = 1.00) but a decrease in taxi use, the second most common method of transportation to SRRH (43.7% vs. 36.4%, *p* value = 0.01). Ambulance utilization decreased from 2.5% prior to the pandemic to 0.8% during the pandemic (*p* value = 0.03). There was no difference in the travel time between cohorts.

**Table 2:** Travel method and time for general surgery patients arriving to SRRH (*n* = 1547^a^)

Pre-hospital characteristic	Pre-pandemic (*n* = 1159)	Pandemic (*n* = 388)	*p *value
*Transport method (n* = *1501)*			
Boda	552 (49.3%)	222 (58.1%)	< 0.01*
Taxi	488 (43.6%)	139 (36.4%)	0.01*
Private car	37 (3.3%)	12 (3.1%)	1.00
Ambulance	28 (2.5%)	3 (0.8%)	0.03*
Bicycle	0 (0.0%)	0 (0.0%)	–
Walking	13 (1.2%)	6 (1.6%)	0.60
Bus	1 (0.1%)	0 (0.0%)	1.00
*Travel time (n* = *1531)*			
Greater than 4 h	12 (1.0%)	3 (0.8%)	0.77
Within 4 h	1135 (99.0%)	381 (98.2%)	0.77
Within 2 h	1045 (91.1%)	349 (90.9%)	0.92
Within 1 h	609 (53.1%)	219 (57.0%)	0.19

*p-value < 0.05

^a^Variables had missing data; hence, the total n differs for each variable. Missing data were excluded from the analysis

### Surgery characteristics

Surgical characteristics and case volume were recorded and compared. There was no difference in median patients recorded per month (32.5 vs. 25.0, *p *value = 0.21) (Table 3). Similarly, there was no difference in median number of elective (24.5 vs. 20.0, *p *value = 0.16) or emergent (6.0 vs. 6.0, *p *value = 0.36) surgeries per month.

**Table 3:** Surgical and hospital course characteristics of general surgery patients arriving to SRRH (*n* = 1547^a^)

In-hospital characteristics	Pre-pandemic (*n* = 1159) (median [IQR])	Pandemic (*n* = 388) (median [IQR])	*p* value
Total patients per month (*n* = 1547)	32.5 [18.8–46.0]	25.0 [17.8–34.5]	0.21
Length of stay (days) (*n* = 1517)	4.0 [2.0–9.0]	5.0 [2.0–10.0]	0.73
Type of surgery (*n* = 1544)			
Elective per month	24.5 [14.3–37.5]	20.0 [14.0–23.0]	0.16
Emergent per month	6.0 [3.3–8.0]	6.0 [4–12]	0.36
Most Frequent operations per month (*n* = 1547)			
Exploratory laparotomy	5.0 [3.0–7.8]	6.0 [30–10.0]	0.29
Incision and drainage of soft tissue	4.0 [1.3–5.0]	2.0 [1.0–3.0]	0.04*
Inguinal hernia repair	3.0 [1.0–5.0]	4.0 [2.0–8.0]	0.22
Herniotomy	1.0 [0.0–3.8]	2.0 [0.8–2.3]	0.70
Excision/resection of mass	1.5 [0.0–3.0]	0.5 [0.0–1.0]	0.04*
Colostomy	0.0 [0.0–1.0]	0.0 [0.0–1.3]	0.84

*p-value < 0.05

^a^Variables had missing data; hence, the total n differs for each variable. Missing data were excluded from the analysis

The most frequent general surgery operations performed at SRRH pre-pandemic were exploratory laparotomy, soft tissue incision and drainage, inguinal hernia repairs, herniotomies, excision/resection of masses, and colostomy creation. These remained the most frequent surgeries during the pandemic; however, less patients per month underwent incision and drainage (4.0 vs. 2.0, *p *value = 0.04) and excision/resection of masses (1.5 vs. 0.5, *p* value = 0.04).

### Delays in surgical care

The COVID-19 pandemic was associated with increased surgical care delays. Prior to the pandemic, 22.6% of patients were reported to have had a care delay (Table 4). During the pandemic, 46.6% of patients faced delay (*p *value < 0.01).

**Table 4:** Provider-reported delays in general surgical care at SRRH prior to and during the COVID-19 pandemic (*n* = 1547^a^)

Delays in Surgical Care	Pre-pandemic (*n* = 1159)	Pandemic (*n* = 388)	*p* value
Total	262 (22.6%)	181 (46.6%)	< 0.01*
Infrastructure delay	196 (16.9%)	180 (46.3%)	< 0.01*
Theater space	192 (16.6%)	180 (46.4%)	< 0.01*
Electricity	6 (0.5%)	0 (0.0%)	0.35
Water	0 (0.0%)	0 (0.0%)	1.00
Personnel delay	36 (3.1%)	18 (4.6%)	0.15
Lack of anesthetist	15 (1.3%)	1 (0.3%)	0.09
Lack of surgeon	18 (1.6%)	17 (4.4%)	< 0.01*
Delay in diagnosis or consult	6 (0.5%)	0 (0.0%)	0.35
Equipment Delay	51 (4.4%)	2 (0.5%)	< 0.01*
Blood products	3 (0.3%)	2 (0.5%)	0.60
Medication	4 (0.3%)	0 (0.0%)	0.58
Sterile linen	15 (1.3%)	0 (0.0%)	0.02*
Surgical Instrument	7 (0.6%)	0 (0.0%)	0.20
Sutures	27 (2.3%)	0 (0.0%)	< 0.01*
Other cause of delay	8 (0.7%)	1 (0.2%)	0.46

*p-value < 0.05

^a^Some patients incurred multiple delays (*n* = 445)

Infrastructure deficits were the most common cause of delay prior to the pandemic and significantly increased during the pandemic (16.9% vs. 46.3%, *p *value < 0.01). This was primarily due to lack of operating space (16.6% vs. 46.4%, *p *value < 0.01). Deficits in electricity and water did not cause delays during the pandemic (*p* value = 0.35 and 1.00, respectively). Personnel shortages was the next largest contributor to delays, demonstrating an increase in delays due to a lack of surgeon (1.6% vs. 4.4%, *p*-value < 0.01).

Delays due to equipment decreased during the pandemic (4.4% vs. 0.5%, *p*-value < 0.01). Shortages of sterile linen and sutures were the largest factors in equipment delays pre-pandemic; however, these delays significantly decreased during the pandemic (1.3% vs. 0.0%, *p*-value < 0.01 and 2.3% vs. 0.0%, *p*-value < 0.01, respectively).

**Fig. 2:**
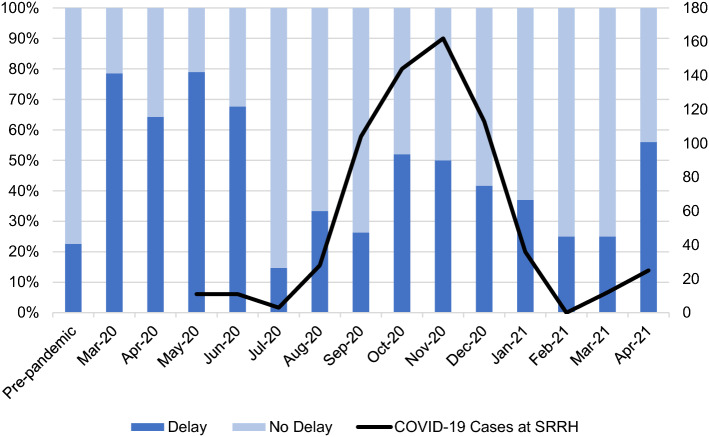
Proportion of patients with delays in surgical care at SRRH and number of reported COVID-19 cases at SRRH by pandemic month (*n* = 1544^a^). ^a^Patients admitted in May 2021 were excluded from analysis due to incomplete data collection

Rates of delay were significantly higher in the first months of the pandemic (March to June 2020), with 64.3% to 78.9% facing delays (Fig. 2). In July 2020, delays decreased closer to pre-pandemic levels at 14.7%. Another wave of worsening delays was observed from August to December 2020 (ranging from 26.3% to 52.0%) which correlated with 13 times increase in COVID-19 cases at SRRH compared to the previous three months. Subsequently, delays decreased from January to March 2021 (25.0% to 37.0%) and COVID-19 cases decreased by 85.5%. April 2021 saw increased delays to 56.0% with a 56.2% increase in COVID-19 cases.

### Adverse events

Lastly, adverse events, including death and complications, were compared. A higher percentage of patients experienced adverse events during the pandemic (5.7% vs. 7.7%, *p* value = 0.18), including more death (2.8% vs. 4.6%, *p* value = 0.10) and complications (5.2% vs. 7.0%, *p* value = 0.20), but did not reach statistical significance (Table 5).

**Table 5:** Adverse events seen in general surgical patients at SRRH prior to and during the COVID-19 pandemic (*n* = 1547^a^)

Outcome	Pre-pandemic (*n* = 1159)	Pandemic (*n* = 388)	*p* value
Total adverse events	66 (5.7%)	30 (7.7%)	0.18
Death	33 (2.8%)	18 (4.6%)	0.10
Complications	60 (5.2%)	27 (7.0%)	0.20

^a^Adverse events is a composite variable of death and complications. Some patients incurred a surgical complication prior to death

## Discussion

This study assessed how COVID-19 affected surgical capacity of a regional referral hospital in Soroti, Uganda. As of January 28, 2022, Uganda had 161,421 confirmed COVID-19 cases causing 3522 deaths [15], representing 3600 infections and 79.4 deaths per million people [16]. This is less than many countries in Africa [16] and far less than the United States, which incurred 225,400 infections per million people by that date [17], potentially attributable to prior experience with pandemics, clear leadership structure, an experienced workforce, strong lockdown policies, and responsive population [18]. With such practices, Uganda has been considered a role model in pandemic containment [19]. Geographically, our study showed decreased proportion of patients from districts further from Soroti like Amuria and an increase in patients who live locally, likely due to strong lockdown restrictions in Uganda. This was expected, given policies blocked public transport and patients often required letters from local leaders prior to crossing districts. However, we demonstrated no change in how long patients experienced symptoms before arriving at SRRH and no change in number of surgical cases during the pandemic, suggesting patients still accessed surgical care perhaps due to relatively good pandemic control.

Reductions in emergency surgery during the COVID-19 pandemic was seen in a variety of different countries [3–5, 20]. In contrast, at SRRH, there was a stable number of emergency and elective surgeries per month prior to and during the pandemic. This is likely due to the relatively good control of COVID-19 in Uganda resulting in patients with emergent surgical conditions still being able to reach care.

The surgical landscape at SRRH was still significantly altered during the COVID-19 era. At SRRH, the proportion of patients experiencing a delay in surgical care more than doubled during the pandemic, signifying this already-tenuous surgical system struggled to cope. In a multi-center study examining hospital readiness during the Ebola and COVID-19 eras, hospitals in Uganda reported multiple barriers to safe surgical care including inability to test all patients for COVID-19, lack of operating theaters to dedicate to COVID-positive patients, inability to regularly disinfect surfaces, inability to reduce staff to protect human resources, and low capacity to provide psychological support to staff who are working in crisis. Overall, none of the forty-seven hospitals in Uganda surveyed met the standards to be considered ready for safe surgical care provision [21]. Many similar factors also caused surgical delay in this study at SRRH. These barriers to safe surgical practices could have detrimental consequences but remain inadequately addressed.

Operating space deficits was the largest contributor to delays in surgical care prior to the pandemic and over twice as many patients faced this delay during the pandemic. In settings like SRRH where operating space is substantially limited, additional strain from pandemic protocols ensuring operating room safety can cause significant reductions in surgical capacity. Provisions for safe surgical care are often neglected in epidemic resource allocation, but financial investment in operating space is an essential component of ensuring safe healthcare delivery in current and future epidemics.

Surgical delays due to lack of surgeons also increased during the COVID-19 pandemic at SRRH. Senior surgeons at SRRH were infected by COVID-19 and, with only two general surgeons at SRRH, having one surgeon unable to work impacted surgical delivery. COVID-19-related factors have been reported in prior studies that could significantly impact the surgeon workforce in LMICs. Providers in LMICs have reported disproportionately received less training in COVID-19 protocols; less access to N95s, gloves, and eye protection; and were more likely to feel unsafe performing their clinical duties compared to providers in HICs [22]. Surgeons and surgical trainees globally were also redeployed outside of their specialties to combat the pandemic, reducing the surgical workforce [23–25]. The future of the surgical workforce has also been impacted, with trainees reporting less operating room exposure, higher focus on service rather than learning, and relative inadequacy of virtual teaching [26]. Sandal et al. [27] demonstrated trainees from LMICs felt stronger negative impact on their training due to COVID-19 compared to trainees from HICs. With current and future surgeons at risk, investments protecting the health and safety of healthcare workers and ensure the future of the workforce in LMICs are necessary.

Deficits in equipment were also a cause of delay in our setting. At SRRH, equipment shortages are primarily due to financial constraints of being a public hospital and efforts to increase the number of instruments from the Ugandan Ministry of Health have been unsuccessful thus far. Sterilization of existing instruments in between procedures can present a significant delay; therefore, procuring more equipment could be a significant asset to improving timely surgical care in our setting.

In this study, waves of delays appear to correlate with waves of COVID-19 cases in Uganda and SRRH. The period of our study demonstrating the most delays was the onset of the pandemic, from March to June 2020. Reports of COVID-19 cases in Uganda [16] and SRRH were low during this time due to limited testing in Uganda until late April [28] and no testing at SRRH until late May 2020. The sharp spike in delays at SRRH was likely due to the profound shift in all global systems, including the surgical system. The subsequent surge in delays from August to December 2020 was reflected in a surge of COVID-19 at SRRH. Though direct causality cannot be assessed, correlation between waves of surgical delays and COVID-19 as well as previously discussed evidence of pandemic-related barriers to surgical delivery [21] suggests the strain from COVID-19 influences surgical capacity at SRRH.

### Limitations

Several limitations were noted for this study. As a single-center study, the ability to generalize results is limited. Delays were defined by surgeons/providers and could be susceptible to bias. Times of arrival and operation were not captured, which limits our analysis of delay severity. Lockdown and infection control measures also prevented research assistants from capturing all surgical cases and deaths during the pandemic. Cohorts had large differences in size, which could make comparisons difficult; however, the degree of statistical difference provides a convincing argument that differences represent real phenomena.

## Conclusions

The surgical landscape at Soroti Regional Referral Hospital (SRRH) in Soroti, Uganda faced significant alterations during the COVID-19 pandemic. Patients at SRRH were more than twice as vulnerable to having a delay in surgical care, particularly from lack of operating space and surgeons. Correlation between care delays and COVID-19 cases at SRRH suggests the strain from COVID-19 influenced surgical capacity at SRRH. Bolstering surgical systems when not in crisis and providing provisions for safe surgical practice in current and future epidemic resource allocations is necessary to strengthen the overall healthcare system. Financial investment in theater space and the surgical workforce and development of tiered triaging protocols for surgery allocation is necessary at SRRH in anticipation of future crises.

## References

[1] Lai CC, Wang CY, Wang YH, Hsueh SC (2020). Global epidemiology of coronavirus disease 2019 (COVID-19): disease incidence, daily cumulative index, mortality, and their association with country healthcare resources and economic status. Int J Antimicrob Agents.

[2] Chang AY, Cullen MR, Harrington RA, Barry M (2020). The impact of novel coronavirus COVID-19 on noncommunicable disease patients and health systems: a review. J Intern Med.

[3] Dick L, Green J, Brown J, Kennedy E (2020). Changes in emergency general surgery during Covid-19 in Scotland: a prospective cohort study. World J Surg.

[4] Riechert M, Sartelli M, Weigand MA, Doppstadt C (2020). Impact of the SARS-CoV-2 pandemic on emergency surgery services—a multi-national survey among WSES members. World J Emerg Surg..

[5] O’Connell RM, Khan MA, Amir M, Bucheeri M, et al (2020) The impact of COVID-19 on emergency general surgery admissions and operative volumes: a single centre experience. Surgeon. 2021 Oct; 19(5): e207–e212 10.1016/j.surge.2020.09.013 PMC7674128 33257272

[6] Meara JG, Leather AJ, Hagander L, Alkire BC (2015). Global surgery 2030: evidence and solutions for achieving health, welfare, and economic development. Lancet.

[7] Tran TM, Fuller AT, Butler EK, Makumbi F (2017). Burden of surgical conditions in uganda: a cross-sectional nationwide household survey. Ann Surg.

[8] Linden AF, Sekidde FS, Galukande M, Kowlton LM (2012). Challenges of surgery in developing countries: a survey of surgical and anesthesia capacity in Uganda's public hospitals. World J Surg.

[9] Albutt K, Punchak M, Kayima P, Namanya DB (2018). Access to safe, timely, and affordable surgical care in Uganda: a stratified randomized evaluation of nationwide public sector surgical capacity and core surgical indicators. World J Surg.

[10] Ozgediz D, Galukande M, Mabweijano J, Kijjambu S (2008). The neglect of the global surgical workforce: experience and evidence from Uganda. World J Surg.

[11] Bellamkonda N, Motwani G, Wange AH, De Boar C (2020). Cost-effectiveness of exploratory laparotomy in a regional referral hospital in eastern Uganda. J Surg Res.

[12] Harris PA, Taylor R, Minor BL, Elliot V (2019). The REDCap consortium: Building an international community of software platform partners. J Biomed Inf.

[13] Starr S, Kim W, Oke R, Carvalho M (2022). The Third Delay in General Surgical Care in a Regional Referral Hospital in Soroti Uganda. World J Surg.

[14] IBM Corp. Released 2019. IBM SPSS statistics for windows, version 27.0. Armonk, NY: IBM Corp

[15] WHO Coronavirus (COVID-19) Dashboard. World Health Organization, https://covid19.who.int/

[16] Covid-19: Ministry of Health. COVID-19 | ministry of health, Ugandan ministry of health, https://www.health.go.ug/covid/. Accessed 22 Mar 2020

[17] CDC Covid Data Tracker. Centers for disease control and prevention, centers for disease control and prevention, https://covid.cdc.gov/covid-data-tracker/#datatracker-home

[18] Kitara DL, Ikoona EN (2020). COVID-19 pandemic Uganda's story. Pan Afr Med J.

[19] Sarki AM, Ezeh A, Stranges S (2020). Uganda as a role model for pandemic containment in Africa. Am J Public Health.

[20] Rashdan M, Al-Taher R, Al-Qaisi M, Khrais I (2021). The impact of the Covid-19 pandemic on emergency surgery in a tertiary hospital in Jordan. A cross sectional study. Ann Med Surg (Lond).

[21] Sikakulya FK, Ssebuufu R, Longombe AA, Okedi XF (2021). Health facilities’ readiness for safe surgical care provision in Uganda and the eastern democratic republic of Congo during Ebola and COVID-19 era. BMC Health Serv Res.

[22] Starr N, Capo-Chichi N, Moore J, Shreckengost CH (2021). Perioperative provider safety in low- and middle-income countries during the COVID-19 pandemic: a call for renewed investments in resources and training. Ann Surg.

[23] DePeralta D, Hong A, Choy C, Wang J (2020). Primer for intensive care unit (ICU) redeployment of the noncritical care surgeon: Insights from the epicenter of the coronavirus disease 2019 (COVID-19) pandemic. Surgery.

[24] Joseph EA, Martins RS, Tariq J, Namrah A (2021). Surgical education and training during the COVID-19 pandemic: strategies and solutions for pakistan. J Pak Med Assoc.

[25] Ferrara F, Galmarini V, Tosco P, Molinari G (2021). Redeployment of specialist surgeons in the COVID-19 pandemic in a general hospital: critical issues and suggestions. Acta Biomed.

[26] Yiasemidou M (2021). The impact of COVID-19 on surgical training: the past, the present and the future. Indian J Surg.

[27] Sandal S, Boyarski BJ, Cantarovich M (2021). The higher impact of the COVID-19 pandemic on resident/fellow training in low- and middle-income countries. Transpl Int.

[28] Ritchie H, Mathieu E, Rodes-Guirao L, Appel C, et al (2020) Uganda: coronavirus pandemic country profile. Our world in data, University of Oxford, https://ourworldindata.org/coronavirus/country/uganda. Accessed 5 Mar 2020

